# Study of factors influencing preoperative detection of alveolar antral artery by CBCT in sinus floor elevation

**DOI:** 10.1038/s41598-020-67644-9

**Published:** 2020-07-02

**Authors:** Pablo Varela-Centelles, María Loira, Antonio González-Mosquera, Amparo Romero-Mendez, Juan Seoane, María José García-Pola, Juan M. Seoane-Romero

**Affiliations:** 1 C.S. Praza Do Ferrol. EOXI Lugo, Cervo, e Monforte de Lemos, Galician Health Service, 27001 Lugo, Spain; 2 Department of Surgery and Medical-Surgical Specialities, School of Medicine and Dentistry, University of Santiago de Compostela, 15782 Santiago de Compostela, A Coruña Spain; 3 Department of Surgery and Medical-Surgical Specialities, School of Medicine, University of Oviedo, 33006 Oviedo, Spain

**Keywords:** Oral anatomy, Cone-beam computed tomography

## Abstract

This study aimed at assessing the prevalence of alveolar antral artery (AAA) detection by CBCT, its related variables, and at describing explanatory models useful in surgical planning, by retrospective evaluation of CBCT explorations. The modelling of the probability for detecting AAA was undertaken using logistic generalized additive models (GAM). The capacity for discriminating detection/no detection was assessed by receiver operating characteristic curves. A total of 466 sinuses were studied. Univariate models showed detection probability was linked to sinus width and thickness of the lateral bony wall, together with the shape and height of the osseous crest. AAA detection probability increased steadily until the thickness of the bony wall reached 6 mm. Multivariate models resulted good discriminators for AAA detection, particularly for females, showing an area under the curve (AUC) of 0.85. Models considering patients altogether, and those including only males offered slightly lower values (AUC = 0.79). The probability of AAA detection by CBCT was influenced by gender (higher in males and for narrow sinuses) and increases with the thickness of the sinus lateral bony wall and the height of the residual alveolar ridge. Besides, and particularly for women, the thickness of the ridge at the basal level seems to improve the explanatory model for AAA detection.

## Introduction

Maxillary sinus floor elevation (SFE) by lateral approach is a safe and predictable procedure for augmenting bone tissue volume for implant placement^1^, but certain potential complications have to be taken into account intra-operatively (Schneider's membrane perforation, haemorrhage from the alveolar antral artery (AAA), osteomeatal complex obstruction) and post-operatively (sinus congestion, graft mobility, acute sinusitis, and cyst formation)^2^.

Accidental bleeding secondary to surgical damage of AAA is the second most frequent complication of SFE^2,3^. In fact, up to 20% of major bleeding events are due to accidental AA impairment^4,5^. These events may result in suspension of the surgery, a slower surgical procedure, reduction of blood supply, mobilisation of the graft, as well as in an increase in the risk for membrane perforations^2^.

The AAA is an anastomosis of the posterior superior alveolar artery (PSAA) and the infraorbital artery (IOA) which has been repeatedly identified by dissection in 100% of the lateral sinus walls of cadavers^6–10^. The vessel can follow either a straight or a U-shaped course in the anterolateral wall of the sinus, reaching its closest point to the alveolar crest at the site of the first or second molar^11,12^. The AAA also maintains a varying relationship with the sinus wall, being usually completely intraosseous and rarely (< 8%) more superficial (under the periosteum) on the lateral wall^3,12^. These circumstances make AAA identification during surgical planning particularly important for avoiding undesired complications^13^.

Although cone beam computed tomography (CBCT) has proved better than conventional computed tomography (CT) at identifying AAA^3^, it cannot be always detected particularly when its diameter is smaller than 0.5 mm or the vessel is in an intrasinusal location or on a superficial position^11,14^. In this vein, the pooled prevalence of AAA detection by CBCT reported in a recent meta-analysis was 78.12% (95%CI: 61.25–94.98)^3^, although some of the original investigations had potential biases and poor internal validity (lack of control for potential confounding factors, such as age, gender, or ethnicity)^5,14,15^.

Recent reports have described the location of AAA in terms of height of the alveolar crest and the diameter of the AAA^16,17^, but produced no predictive models explaining the detection of the AAA in the lateral wall of the maxillary sinus.

Considering the lack of studies on AAA detection controlling for the aforementioned confounding factors, the aims of this investigation were to render information on the prevalence of AAA detection by CBCT, to study the variables related to its detection while controlling for potential confounding factors, and also to describe explanatory models to help in surgical planning for these patients.

## Results

A total of 466 maxillary sinuses were studied, 198 males (42.5%) and 268 females (57.5%), whose median age was 57 years old (IQR: 49.25–65). CBCT explorations identified the AAA in 240 sinuses (51.5%), with a median of the distance from AAA to the sinus floor of 7.10 mm (IQR: 5.20–9.70), and a median distance from AAA to the alveolar crest of 14.90 mm (IQR: 12.20–17.50). The median width of the sinus lateral wall was 1.80 mm (IQR: 1.5–2.5). AAA mostly described a fully intraosseous course within this wall (48.7%) with a diameter ranging from 1 to 2 mm in most cases (67.5%).

The predominant subsinusal bone resorption patterns were the “well-rounded” and “flat ridge” forms (class II: 7.2%; class III: 38.3%; class IV: 13.0%; class V: 24.2%; class VI: 17.3%), with a ridge height of 7.10 mm (IQR: 4.9–9.7) (Table 1).

**Table 1:** Main features of the sample studied.

Variables	n	(%)
**Gender**		
Male	198	42.48
Female	268	57.52
**AAA detection**		
No	226	48.49
Yes	240	51.51
**AAA position**		
Fully intraosseous	117	48.75
Superficial	8	3.33
Intrasinusal	115	47.91
**Pattern of edentulousness**		
Fully edentulous maxilla	59	12.6
Subsinusal edentulousness	329	70.6
Upper first molar missing	78	16.7

	Mean	Median (IQR)
Age (years)	56.88	57.00 (49.25–65.00)
Thickness of the lateral sinus wall (mm)	2.31	1.80 (1.50–2.50 )
Height of the residual alveolar ridge (mm)	7.44	7.10 (4.90–9.70)
Width of the residual alveolar ridge (basal level) (mm)	10.06	10.10 (8.20–11.90)
Width of the residual alveolar ridge (crestal level) (mm)	6.62	6.40 (4.70–11.90)
Maxillary sinus width (mm)	12.60	12.40 (10.05–14.80)

Univariate models have shown a probability for AAA detection linked both to the sinus width and to the thickness of the lateral bony wall, as well as to the shape and height of the osseous crest. The probability for AAA detection increased steadily until the thickness of the bony wall reached 6 mm. Beyond this point, the probability diminished. A decreasing trend in the chances for detection was also observed when the sinus width increased. Conversely, the chances for identifying the AAA increased with the height of the residual crest: post-extraction, rounded crests were significantly linked to AAA detection when compared to flat and depressed ridges (Table 2).

**Table 2:** Univariate logistic models.

Univariate linear logistic models	Estimate (ß)	Standard Error	Z value	*p*-value	Degrees of freedom (df)	Model's Chi Square	Model's *p*-value
**Gender**					1	2.79	0.09
Intercept	0.75	0.16	4.51	6.41e−06			
Female	− 0.36	0.21	− 1.67	9.44e−02			
**Pattern of edentulousness**					2	0.53	0.06
Intercept	0.15	0.24	0.61	0.53			
Fully edentulous maxilla versus upper first molar missing	0.60	0.39	1.50	0.13			
Subsinusal edentulousness versus upper first molar missing	0.66	0.28	2.35	0.01*			
**Classification of posterior maxilla**					4	16.96	0.00
Intercept	− 0.09	0.24	− 0.37	0.70			
Class IV versus Class VI	0.54	0.38	1.42	0.15			
Class V versus Class VI	0.38	0.32	1.16	0.24			
Class II versus Class VI	1.34	0.52	2.56	0.01**			
Class III versus Class VI	1.11	0.31	3.58	0.000***			
Class V versus Class IV	− 0.16	0.36	− 0.46	0.64			
Class II versus Class IV	0.79	0.54	1.45	0.14			
Class III versus Class IV	0.56	0.34	1.63	0.10			
Class II versus Class V	0.96	0.50	1.89	0.05			
Class III versus Class V	0.73	0.28	2.59	0.00***			
Class III versus Class II	− 0.22	0.50	− 0.45	0.64			

Univariate GAM logistic models	Explained deviance (%)	Effective degrees of freedom (edf)	Model's Chi square	Model's *p*-value
Age (years)	0.85	1.67	2.53	0.18
Thickness of the lateral sinus wall (mm)	5.16	1.92	22.57	2.59e−05***
Height of the residual alveolar ridge (mm)	1.20	1.29	6.40	0.04*
Width of the residual alveolar ridge (basal level) (mm)	0.99	1.75	4.41	0.14
Width of the residual alveolar ridge (crestal level) (mm)	1.26	1.79	3.64	0.13
Maxillary sinus width (mm)		1.00	34.69	3.85e−09 ***

*p < 0.05; **p < 0.01; ***p < 0.001.

The best multivariate model revealed an explained deviance of 21%, and included gender, pattern of edetulousness, sinus width, thickness of the sinus lateral bony wall, and shape and height of the ridge, with α representing the intercept, β_i_ representing the beta coefficients of the covariates in the parametric part of the model, f_i_ (unknown) smooth functions of continuous covariates, and been Logit the link function of the logistic model.

(Logit {P (Detection|X_i_)} = α + β_1_ gender + β_2_ type of edentulousness + β_3_ ridge shape + f_1_ (ridge height) + f_2_ (thickness of lateral bony wall) + f_3_ (sinus width)), where f_1_ to f_3_ represent smooth functions of covariates.

**Figure 1:**
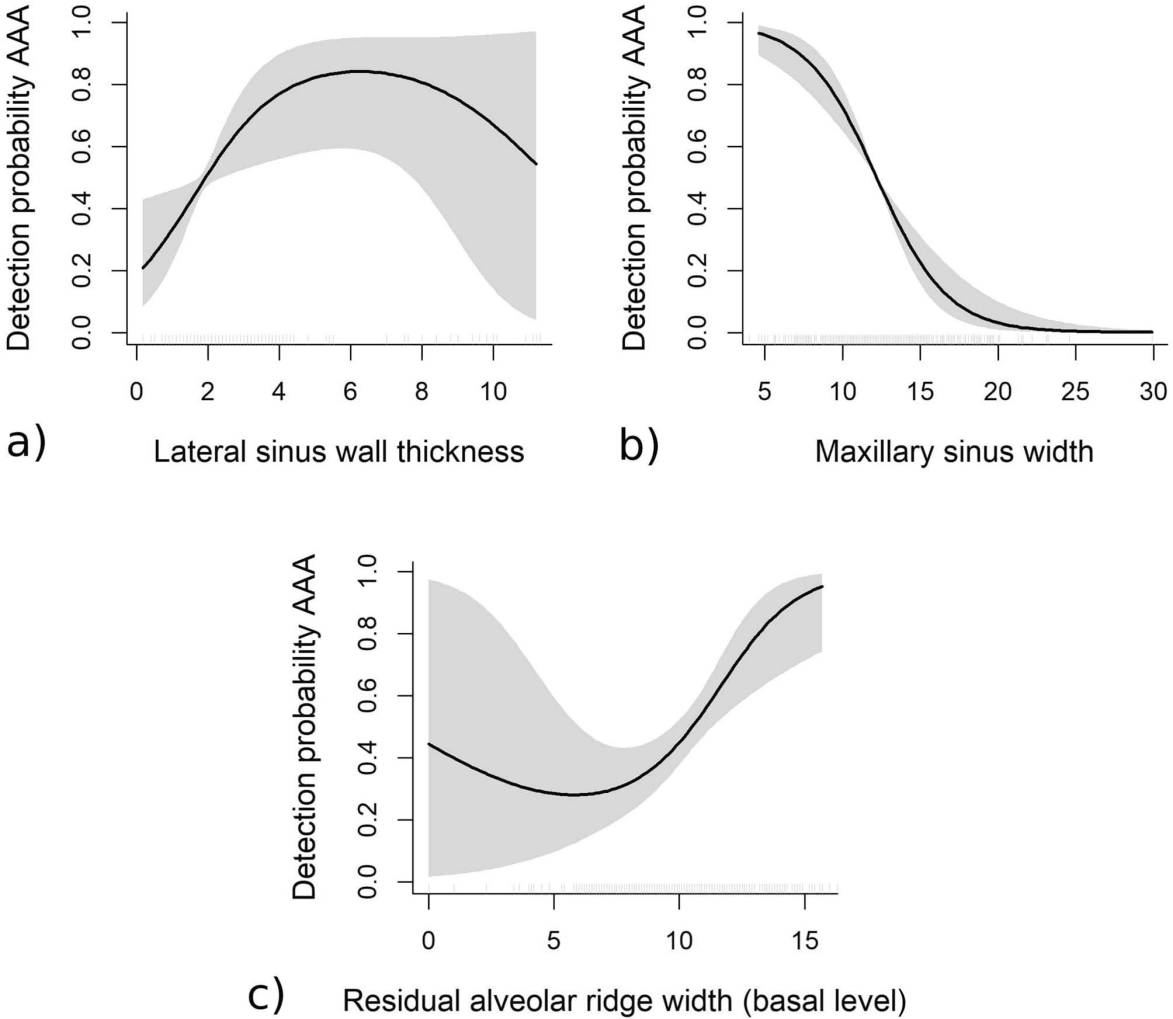
Detection probability for female patients (shaded grey area representing 95% point-wise confidence band). (**a**) Effect of lateral sinus wall thickness on detection probability AAA; (**b**) Effect of maxillary sinus width on detection probability AAA; (**c**) Effect of residual alveolar ridge on detection probability AAA.

When adjusting for gender, the best multivariate model for females was (Logit { P (Detection | X_i_)} = α + β_1_ type of edentulousness + β_2_ ridge shape + f_1_ (width of the ridge at the basal level) + f_2_ (ridge height) + f_3_ (thickness of the lateral bony wall) + f_4_ (sinus width)), which explained a deviance of 31.2% (Table 3). In women, rounded crests (class III) are linked to a significantly higher probability for AAA detection when compared to other resorption patterns. Besides, the width of the ridge at the basal level resulted to be significantly associated to artery detection, and this chance is increased for values over 5 mm. A similar situation occurred when the thickness of the lateral bony wall was considered, where the probability for AAA detection increased with thicknesses up to values about 4 mm, to decrease beyond this point, although the confidence interval was too wide to consider this effect as significant (Fig. 1).

**Table 3:** Multivariate model.

Multivariate model by gender	Female (percentage of deviance explained by the model = 31.2%)				Male (deviance percentage explained by the model = 22%)			
Parametric part of the model	Estimate (ß )	SE	Z value	*p*-value	Estimate (ß )	SE	Z value	*p*-value
**Pattern of edentulousness**								
Intercept	− 0.69	0.77	− 0.90	0.36	− 0.91	0.72	− 1.27	0.20
Fully edentulous maxilla versus upper first molar missing	1.16	0.85	1.36	0.17	1.54	0.67	2.29	0.02**
Subsinusal edentulousness versus upper first molar missing	0.88	0.47	1.86	0.06	1.82	0.54	3.33	0.00**
Subsinusal edentulousness versus fully edentulous maxilla	− 0.27	0.74	− 0.36	0.71	0.28	0.54	0.51	0.60
**Classification of posterior maxilla**								
Class IV versus Class VI	0.19	0.85	0.22	0.82	0.63	0.84	0.75	0.45
Class V versus Class VI	0.43	0.71	0.60	0.54	0.23	0.63	0.37	0.71
Class II versus Class VI	− 0.05	1.07	− 0.05	0.95	3.05	1.33	2.29	0.02**
Class III versus Class VI	1.57	0.78	1.99	0.04*	1.46	0.78	1.86	0.06
Class V versus Class IV	0.23	0.69	0.34	0.73	− 0.39	0.76	− 0.51	0.60
Class II versus Class IV	− 0.25	0.99	− 0.25	0.79	2.42	1.33	1.81	0.06
Class III versus Class IV	1.37	0.70	1.95	0.05*	0.82	0.74	1.11	0.26
Class II versus Class V	− 0.49	0.87	− 0.56	0.57	2.82	1.27	2.21	0.02**
Class III versus Class V	1.13	0.50	2.24	0.02*	1.22	0.65	1.87	0.06
Class III versus Class II	1.63	0.82	1.96	0.04*	− 1.59	1.21	− 1.30	0.19

Flexible part of the model (smooth terms)	Degrees of freedom (df)	Effective degrees of freedom (edf)	Chi square	Model's *p*-value	Effective degrees of freedom (edf)	Degrees of freedom (df)	Chi square	Model's *p*-value
Thickness of the lateral sinus wall	1.76	1.94	7.01	0.03*	1	1	7.67	0.00**
Height of the residual alveolar ridge	1.80	2.27	5.32	0.11	1	1	3.42	0.06
Maxillary sinus width	1.00	1.00	30.47	3.39e−08***	1	1	8.97	0.00**
Width of the residual alveolar ridge (basal level)	1.76	1.94	11.16	0.00**				

*p < 0.05; **p < 0.01; ***p < 0.001.

Conversely, there was a very clear association between sinus width and AAA detection, where chances for artery identification markedly diminished as sinus width increased, being just about zero for widths over 20 mm (Fig. 1, Table 3).

**Figure 2:**
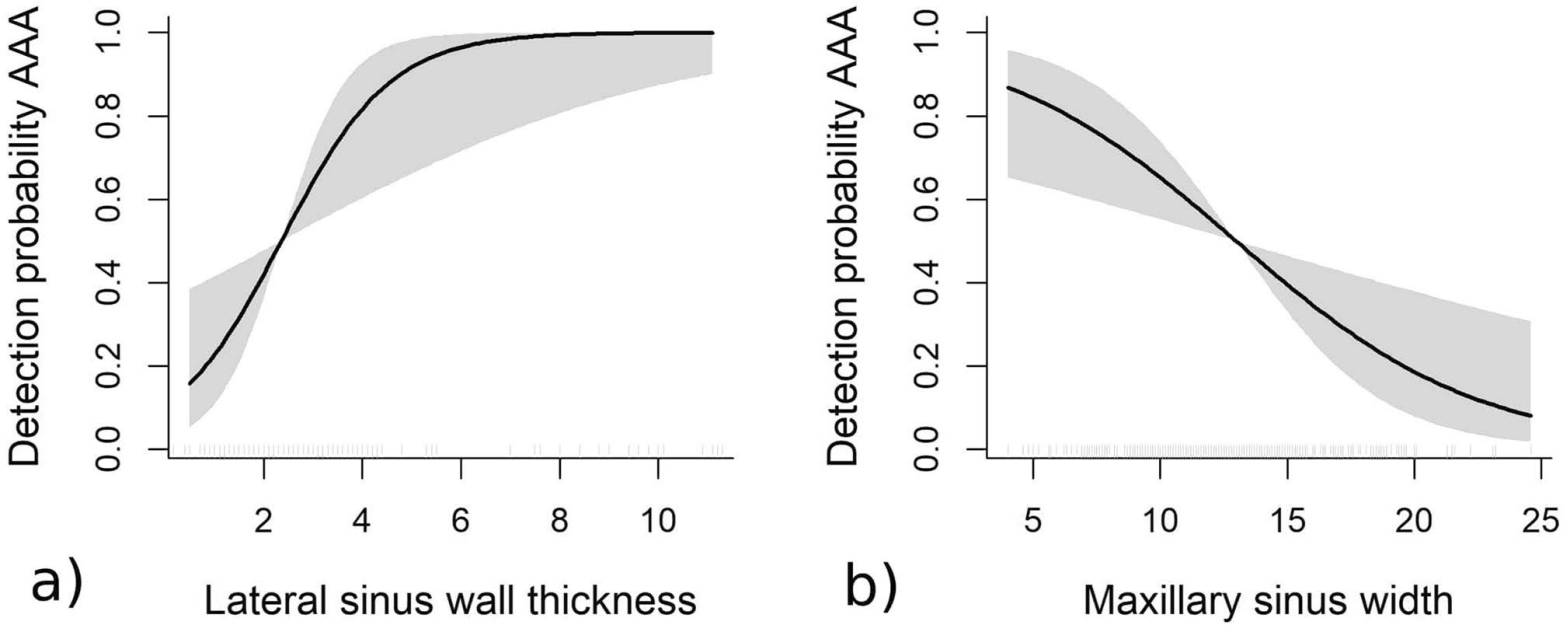
Detection probability for male patients (shaded grey area representing 95% point-wise confidence band). (**a**) Effect of lateral sinus wall thickness on detection probability AAA; (**b**) Effect of maxillary sinus width on detection probability AAA.

For males, the best model explained a deviance of 22% and included the pattern of edentulism (Logit {P (Detection |X_i_)} = α + β1 type of edentulousness + β2 ridge shape + f_1_ (ridge height) + f_2_ (thickness of the lateral bony wall) + f_3_ (sinus width)). Thus, the chances for AAA detection are higher for fully edentulous patients, when compared to those with only the first upper molar missing. As occurred for women, the probability for AAA detection increased along with the width of the sinus lateral bony wall up to values close to 4 mm but showing higher detection chances in male subjects. The width of the sinus also showed an inverse relationship with the chances for AAA detection, but the decrease in probabilities was less sharp than found for females (Fig. 2).

The multivariate models suggested in this study are good discriminators for AAA detection, particularly in female patients, showing an area under the curve (AUC) of 0.85, whereas those models considering patients altogether or including only male patients offered slightly lower values (AUC = 0.79), which are in the limit of what is considered a good discriminator.

## Discussion

Despite the relevance of pre-surgical identification of AAA for SFE and other procedures involving the lateral wall of the maxillary sinus, such as Le Fort I osteotomies or Caldwell-Luc surgeries^18^, very few isolated studies have focused on the determinants of radiologic AAA detection. In this sense, and regardless of the extra-osseous position of the artery, the low detection rates showed by CBCT and CT (62.0%; 95%CI: 46.33–77.71)^3,12^ when compared to anatomical findings (100%)^6–10^ seem to be explained only by the small diameter of the vessel lumen, the technique (CBCT vs CT), and the skills and experience of the observers. Age and gender have also been related with radiological identification of AAA, although this relationship is based upon poorly consistent results^11,15^.

Two papers on AAA detection by CBCT reported important disparities in their chances to identify the vessel (52.8%^12^ to 90%^18^). As both samples report on patients with the same ethnicity, this wide variation seems to be related to factors linked to the CBCT device and to the observer^12,18^. Our results (51.1%) are close to the lower limit of this range in a sample including 57.5% females.

The usual course of AAA is mostly intraosseous within the lateral wall of the sinus^19–23^, followed in our study by the intrasinusal location (between the sinus membrane and the lateral wall). The anatomically described absence of a bony layer between the AAA and the Schneiderian membrane may influence the surgical handling of AAA, particularly during the procedure of membrane detachment^8,21,24^. A third possible situation of the vessel, just underneath the periosteum with a radiologically visible indentation in the lateral bony wall, was the least frequent one (3.3%) in our study, and less prevalent than reported in the literature^20–22^. The AAA can also describe a fully extra-osseous course^2^, but in these cases the artery lies within the flap, and the risk for haemorrhage would be associated to the incision, but not to the actual antrostomy^21,25^.

Information dealing with AAA diameter is usually reported as a categorical variable because the surgical relevance of damaging the vessel is size-dependent^3^. Hence, damage to a small AAA (< 1 mm) (19.5% in our series, 13.9–55.3% in the literature^14,15^ has a negligible surgical impact^11^ but damage to larger arteries may hamper visualization and hinder the surgical procedure^13^. The prevalence of AAA diameters between 1 and 2 mm is reported to range between 22.1 and 64.9%^11,15^. Vessels with diameters exceeding 2 mm, if damaged, are likely to result in bleeding important enough to interfere with the placement of the bony graft, which is a real surgical complication^14^. In our sample, 12.9% fell within this category (4.3 to 21.3% in other case series^3^. Larger vessels (> 3 mm) should be avoided or ligated to prevent severe iatrogenia^21,25,26^.

The relative position of AAA with reference to the alveolar ridge also influences osteotomy. There are wide variations reported in the literature (from 11.2^14^ to 18.1 mm^19^) probably due to variations in the height of the residual crest. Again, our results rank between the reported values (15.2 mm for an average residual ridge height of about 7.7 mm). Thus, pre-surgical awareness of these variables is paramount to adequately design the osteotomy for SFE procedures.

The current study is the first report detailing explanatory models for AAA detection controlling for potential risk factors. The large size of the sample analysed—which is consistent with previous reports both in terms of age and gender distribution and artery diameter and position-, increases the external validity of our investigation. In addition, the fact that all participants in the study were being explored for dental implant treatments makes a potential selection bias unlikely. However, there are certain limitations for this kind of studies related to inter-observer variability (differences in visual observation and interpretation of images) and their experience. In this sense it has been suggested to include a higher number of observers^27,28^ and also that more experienced observers show higher detection rates^29^. In our study, two experienced surgeons scored a high concordance in AAA detection.

Another potential source of variability is the actual CBCT device^28,30^ and, therefore, extrapolation of our results to different technical equipment should be made with caution. In this vein, and when using CBCT for preoperative diagnosis, higher accuracy than the range of half a millimetre cannot be expected^30^, so clinicians should consider this fact when planning their surgeries to prevent overestimating the precision of CBCT examinations^30^. General surgical recommendations for SFE procedures include CBCT evaluation and a careful preparation of the bony window^21^ keeping a safety distance > 1 mm from AAA to avoid arterial damage.

The influence of age in AAA diameter is equivocal^3,11^. Although some groups have described a positive correlation^11,31^, other reports could not link age with the radiological detection or diameter of AAA^15,19,22^ as occurred in the current study.

On the other hand, gender resulted to be an explanatory variable in the multivariate model influencing artery detection. Larger diameters and higher detection rates were found amongst males^15,18,19^, which implies higher chances for intra-operative bleeding for this group^15–20^. The thickness of the sinus lateral bony wall also behaved as an explanatory variable, as larger AAAs were identified in thicker walls^2,19^. Thus, when facing a thick wall, the risk for bleeding should be anticipated^3,27^. However, the use of flexible models showed that this association is not linear, but chances for detection rapidly increase up to 6 mm thickness to decrease beyond this value. The width of the maxillary sinus also influences the surgical difficulty of SFE^32^ and has an effect on the probability for detecting AAA by CBCT. These chances significantly diminish when the distance between the lateral and medial walls of the sinus increase, particularly among women. Although our sample was mostly made of narrow sinuses (< 14 mm width), the AAA was nearly undetectable in wide sinuses (> 20 mm). The probability for detecting AAA is higher in patients with well-rounded ridges of an adequate height. The chances also increase with height for this particular ridge shape.

Bearing in mind the AAA detection rate by CBCT, particularly for vessels > 0.5 mm with potential to produce relevant bleeding if damaged with conventional rotary instruments, a careful surgical planning and the use of piezoelectric instruments are strongly recommended ^26,33^.

## Conclusions

It is concluded that the probability for AAA detection by CBCT is influenced by patient's gender (higher in males and for narrow sinuses) and increases with the thickness of the sinus lateral bony wall and the height of the residual alveolar ridge. Besides, and particularly for women, the thickness of the ridge at the basal level seems to improve the explanatory model for AAA detection. It is suggested to assess and control for these variables in future studies on this topic.

## Methods

A retrospective observational study was undertaken at the Radiology Unit of the School of Medicine and Dentistry of the University of Santiago de Compostela (Spain) with the approval of the university's Ethics Committee (R00002/640) and in full accordance with the Declaration of Helsinki. After obtaining informed consent, CBCT studies undertaken from November 2008 to November 2015 were identified by means of a database manager and selected according to the following inclusion criteria: CBCT explorations performed for surgical planning of implant-supported prosthesis in either maxillary edentulous or subsinusally edentulous patients, as well as for patients scheduled for replacement of a single upper first molar. Exclusion criteria were poor image quality, sinusal disorders, or previous experience of sinus surgery or grafting.

All patients were explored by means of a cone beam CT (I-CAT, 17–19. Imaging Sciences International, 1910 North Penn Toad, Hatfield, USA) with its I-CAT software (Imaging Sciences International) set at a voxel size of 0.3 mm edge-length with 8.9 s of capture time. Patients were instructed to avoid movement and keep their heads positioned to maintain the Frankfurt plane parallel to the horizontal. The position was checked with the device’s alignment lights and a preliminary view. Images were reoriented when needed.

The field of view was adequate to include the maxillary sinus and CBCT images were viewed in a darkened, quiet room using an EIZO FlexScan S2000 LCD monitor (EIZO NANAO Corporation, Hakusan, Japan) display with a luminance of 3,000 cd/m^2^ and contrast ratio 1,000:1. Multiplanar reconstructions with axial, coronal and sagittal images were assessed.

The presence of the AAA canal was identified following the postero-lateral wall of the maxillary sinus using coronal slices. When necessary, navigation through axial and sagittal images was also undertaken.

The area of the upper first molar was selected for the study because it is the most frequently involved in vascular damage events during sinus elevation surgery^25^, and also because of the proximity of the artery to the bony crest at this anatomical region^25^. Each image was read by two observers, expert in Oral Surgery (ML & AG) and each observer reported if AAA was detected or no. Kappa was estimated as 0.923 (95% CI, 0.888 to 0.958). In order to reduce observer-related variability and to increase the reliability of the study, a workshop to standardise the procedures of linear measurement and AAA detection (intrabony canal: well defined bony canals) or partially intraosseous- notch on the outer cortex of the lateral sinus wall or the inner side of the lateral bony wall) was undertaken prior to data collection.

**Figure 3:**
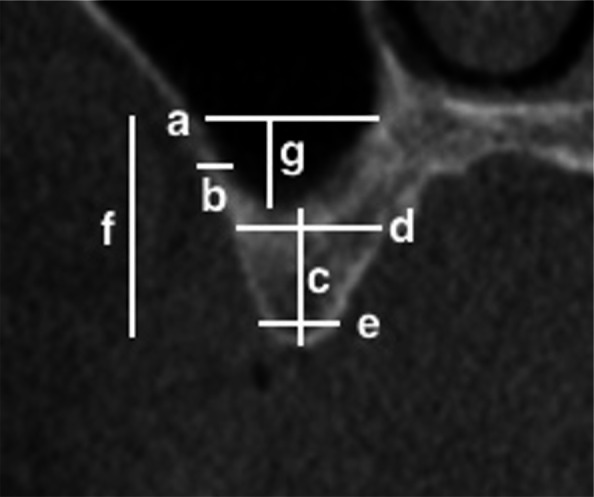
Explanation of linear measurements in CBCT considered in the study. (**a**) AAA; (**b**) Thickness of the lateral sinus wall; (**c**) Height of the residual alveolar ridge; (**d**) Width of the residual alveolar ridge (basal level); (**e**) Width of the residual alveolar ridge (crestal level); (**f**) Distance from AAA to the alveolar crest, (**g**) Distance from the AAA to the sinus floor.

The linear measurement tool of the I-CAT Vision device was used to measure quantitative variables. The relative uncertainty (standard deviation of the measurement divided by its mean and expressed as a percentage from 0 to 100%) was chosen for determining the error of these observations. The variables assessed (with its relative uncertainty) were (Fig. 3): thickness of the lateral sinus wall at 3 mm from the sinus floor (0.25%); height of the residual alveolar ridge: perpendicular distance from the sinus floor to the alveolar crest (0.42%); width of the residual alveolar ridge (basal level) (0.04%); width of the residual alveolar ridge (crestal level) (1.43%); perpendicular distance from the lower border of AAA canal to sinus floor (0%); perpendicular distance from the lower border of the AAA canal to alveolar crest (0%); and maxillary sinus width (distance from medial to the lateral maxillary sinus wall) measured at 15 mm from the ridge crest (0.01%)^5,12,19,20,32^.

Patients' age was also considered as a continuous variable. The main categorical variable studied in this investigation (outcome) was “detection vs no detection of the AAA's bony canal in the lateral sinus wall”. Additional potentially related co-variates, such as gender, pattern of tooth loss (maxillary edentulous, subsinusally edentulous, or absent upper first molar), position of the artery (fully intraosseous; intrasinusal—between the Schneiderian membrane and the sinus bony wall-; or superficial—on the outer cortex of the lateral sinus wall). The type of residual bony crest was classified according to Cawood and Howell^34^ as class II: immediately post extraction; class III: well-rounded ridge; class IV: knife-edge ridge; class V: flat ridge; and class VI: depressed ridge form.

### Statistical analysis

The maxillary sinus was the study unit. The results for categorical variables were expressed as frequencies, whereas quantitative variables were defined by their median and mean as statistics for central trend, and by the inter-quartile range as a spread indicator.

The modelling of the probability for detecting AAA was undertaken using logistic generalized additive models (GAM)^35^. This approach permits modelling the effect of co-variates in the response in a flexible way, as the response variable is dichotomous. Univariate models have been adjusted for each of the independent variables. The method used for model selection between all the candidates was automatic model selection with prediction error criteria based on AIC values. For the model with the lowest prediction error (the selected or best model) the absence of concurvity (the equivalent to collinearity in GAM models) was verified.

The capacity for discriminating between artery detection vs. no detection was assessed by means of receiver operating characteristic (ROC) curves.

All the analyses were carried out with the free software R^36^ using the mgv package^35,37^ for GAM modelling, and ROCR^35,37,38^ for ROC analysis. Inter-observer concordance for two categories was calculated using the R package vcd.

## Supplementary information

Supplementary information

## Data Availability

All data generated or analysed during this study are included in this published article (and its Supplementary Information files).
